# Navigating Misconceptions and Building Understanding: A Deep Dive Into Medical Field Knowledge Gaps Concerning Organ Donation in Saudi Arabia

**DOI:** 10.7759/cureus.91960

**Published:** 2025-09-10

**Authors:** Yousef Alnajjar, Fay Hadi, Saad A Alkarni, Lama S Alahmadi, Rahaf K Alsahli, Shahad Mubarak, Hussain M AlHassan, Mohammed AlSheef, Faris Altom

**Affiliations:** 1 Medicine, Sulaiman Al-Rajhi University, Al Bukayriyah, SAU; 2 Medicine, Jeddah University, Jeddah, SAU; 3 Medicine, University of Bisha, Bisha, SAU; 4 Medicine, Al Rayan College, Madinah, SAU; 5 Medicine, Majmaah University, Majmaah, SAU; 6 Medicine, King Khalid University, Abha, SAU; 7 Trauma, Saudi Red Crescent Authority, Riyadh, SAU; 8 Medicine, King Fahad Medical City, Riyadh, SAU

**Keywords:** attitudes, knowledge gaps, medical students, organ donation, saudi arabia

## Abstract

Background: Although organ donation is a lifesaving medical procedure, there are still obstacles to its widespread use because of misunderstandings, cultural norms, and information gaps, especially among aspiring medical professionals. To increase organ donation rates and close the gap between supply and demand in Saudi Arabia, these obstacles must be removed. The aim of this study was to evaluate Saudi Arabian medical students' knowledge, attitudes, and behaviors about organ donation and determine the variables affecting their opinions.

Methods: From September to December 2024, analytical cross-sectional research was carried out in five different Saudi Arabian areas. Data were gathered using a validated, self-administered computerized questionnaire disseminated across many social media sites using a non-probability convenience sample approach. The survey evaluated general knowledge, attitudes, perceptions, and sociodemographic traits related to organ donation. SPSS version 27 (IBM Corp., Armonk, NY) was used for statistical analysis, and multivariate logistic regression and chi-square tests were used to examine correlations between the variables. A p-value of less than 0.05 was deemed significant.

Results: A total of 473 participants were studied, including 281 (59.4%) female students and 192 (40.6%) male students; most were Saudi nationals (348 (73.6%)), ≤25 years old (425 (89.9%)), and unmarried (430 (90.9%)). Knowledge scores showed 268 (56.7%) good and 205 (43.3%) poor, with employees (31 of 52 (59.6%)) outperforming students (174 of 421 (41.3%)) (p = 0.012). The majority (426 (90.1%)) recognized saving lives as the main goal of organ transplantation, despite misconceptions about brain death and donor eligibility. Men (117 of 192 (60.9%)) were over twice as likely as women (89 of 281 (31.7%)) to support organ donation after death (odds ratio (OR) = 2.26, p = 0.005).

Conclusion: Although Saudi medical students had a reasonable level of general awareness of organ donation, there are still significant information gaps and misunderstandings, especially with regard to legal, procedural, and clinical elements. By addressing these deficiencies with focused educational interventions in medical curricula, future doctors may be better equipped to promote organ donation and have a favorable impact on public opinion.

## Introduction

Organ transplantation has become the preferred intervention for patients with end-stage organ failure [1]. It involves giving human parts, such as organs like kidneys, lungs, and hearts, and even cells and tissues, to a recipient who is in need of restoring a normal functioning way of living [2]. The Saudi Ministry of Health recently stated the importance of considering organ donation and how it affects the quality of life for those who are in need, which leads to lessening the potentiality of death from living or brain-dead donors [3]. Enriching knowledge about such topics must be articulated by medical students to be delivered to patients and their relatives smoothly, in between the words of advice, as a consideration of choice to enlighten others who are in need [3]. Organ dysfunction and failure account for increased mortality rates across most population groups due to the shortfall in the availability of organs [4].

The limited availability of donor organs is significantly influenced by widespread misunderstandings about the legal and procedural aspects of organ donation, as well as religious and cultural obstacles prevalent among the general public. Consequently, it is crucial for medical students, as future doctors and healthcare practitioners, to develop a comprehensive and accurate understanding of organ donation. Their informed perspective is vital in fostering an environment that encourages organ donation and improves procurement rates [5,6].

A 2017 study in Saudi Arabia noted that nearly 6,000 patients are waiting for a donor [7], which signifies the importance of organ donation consideration, especially since the gap between organ needs and donation is large and increasing, making such a need a global issue [8]. Thinking and delivering such topics in a positive attitude encourages students to look up to considering this challenging topic as a choice to be delivered for their future practice with both the donor and the relatives and recipients [9]. Medical students tend to have knowledge gaps regarding organ donation, particularly in legal aspects and differing local and international attributes, for which it is important to raise awareness in this study regarding organ donation [10].

To the best of our knowledge, there has been no study in Saudi Arabia among medical students on misconceptions and understanding of organ donation. Thus, our study aims to assess the knowledge and attitudes of the practice of organ donation processes, to evaluate whether medical students understand organ donation and transplantation processes and to ascertain people's opinions about the topic. While organ donation is still a major topic in medical research to this date, little research has been done to assess the knowledge of organ donation and transplantation among medical students. Organ donation is considered a lifesaving process, and a study on this topic will provide an opportunity to increase public awareness. Hence, this study aimed to evaluate the knowledge, attitudes, and practices related to organ donation among Saudi medical students and identify the factors influencing their understanding and perceptions, highlighting existing gaps and misconceptions.

## Materials and methods

Study design, setting, and population

This study was a descriptive cross-sectional design conducted in Saudi Arabia, specifically across five regions: the Eastern, Western, Central, Northern, and Southern regions. Data collection was carried out between September 2024 and December 2024. Participants included medical students enrolled in Saudi Arabian institutions, while employees from the general community were also surveyed for comparative purposes. Medical students studying outside Saudi Arabia and individuals from non-medical specialties were excluded.

Sample size

The study included a total of 473 participants. The minimum required sample size was calculated as 350 using OpenEpi v.3 (Mini & Nobili, 2017) with a 95% confidence level and a 5% margin of error. All participants met the inclusion criteria of being medical students in Saudi Arabia. For comparative purposes, a group of community employees was also surveyed, while medical students studying outside Saudi Arabia and individuals from non-medical specialties were excluded.

Data collection methods, instruments, and sampling technique

A convenience sampling technique, which is a form of non-probability sampling, was employed to recruit participants. Data were collected using an electronic, self-administered questionnaire distributed to medical students and the community via various platforms, including Twitter, Telegram, WhatsApp, Instagram, and email. Periodic announcements were made to encourage reposting of the questionnaire and maximize reach.

The questionnaire was developed by the authors based on several previous studies and adapted from a validated instrument [8]. Responses from the control group were analyzed separately from those of medical students. The questionnaire underwent pre-testing in a pilot study involving 10 medical students and staff members from the same institution to identify and resolve any ambiguities or misunderstandings. Subsequently, three experts reviewed the questionnaire for validity and reliability. After one week, the questionnaire was re-administered to the same pilot group to assess test-retest reliability.

The final questionnaire comprised 17 items in two sections. The first section contained questions on personal information, while the second included 14 items assessing general knowledge of organ donation, including understanding of legal regulations and the goals of organ transplantation.

Data management and analysis plan

Data were cleaned, managed, and coded using Microsoft Excel 2019 (Microsoft Corporation, Redmond, WA). Statistical analyses were conducted using R (RStudio, version 1.4.1106; RStudio, Inc., Boston, MA). Descriptive statistics, including frequency distributions, were generated, and cross-tabulations were assessed using the chi-square test. Each correct answer in the knowledge section was awarded 1 point to calculate a total knowledge score. Confidence intervals (95% CI) were computed, and multivariate logistic regression was applied. A p-value of <0.05 was considered statistically significant.

Ethical considerations

Ethical approval for this study was obtained from the Institutional Review Board of Riyadh Second Health Cluster (IRB registration number with KACST, KSA: H-01-R-012; IRB registration number with OHRP/NIH, USA: IRB00010471; Federal Wide Assurance Number, NIH, USA: FWA00018774). The study was conducted in accordance with Good Clinical Practice guidelines and the regulations of the Government of Saudi Arabia. All participants were informed of the study objectives, and written informed consent was obtained prior to participation. Participants had the right to withdraw at any time without penalty. Responses were kept confidential, and access to the data was restricted to the authors. Personal information was stored securely and anonymized during analysis.

## Results

A total of 473 participants were included in this study (Table 1). There were 281 (59.4%) female participants and 192 (40.6%) male participants. Most were Saudi nationals (348 (73.6%)), while 125 (26.4%) were non-Saudi. The majority were aged ≤25 years (425 (89.9%)), followed by 26-35 years (30 (6.3%)), 36-45 years (14 (3%)), and >45 years (4 (0.8%)). Regarding marital status, most were single (430 (90.9%)), while 40 (8.5%) were married and three (0.6%) were divorced. In terms of education, 268 (56.7%) held higher education degrees, 203 (42.9%) had completed secondary education, and two (0.4%) had intermediate education. Professionally, 268 (56.7%) were in the medical field, and 205 (43.3%) were in non-medical specialties. Participants were distributed mainly across the Central region (114 (24.1%)), Southern region (112 (23.7%)), and Western region (110 (23.3%)), with additional participants from the Eastern region (99 (20.9%)) and Northern region (38 (8%)). 

**Table 1:** Sociodemographic characteristics of the participants (N = 473)

Category	Subcategory	Counts (number)	Percent of total (%)
Gender	Female	281	59.4
	Male	192	40.6
Nationality	Non-Saudi	125	26.4
	Saudi	348	73.6
Region of residence	Central region	114	24.1
	Eastern region	99	20.9
	Northern region	38	8
	Southern region	112	23.7
	Western region	110	23.3
Age category	≤25	425	89.9
	26-35	30	6.3
	36-45	14	3
	>45	4	0.8
Marital status	Single	430	90.9
	Married	40	8.5
	Divorced	3	0.6
Educational level	Higher education	268	56.7
	Secondary education	203	42.9
	Intermediate education	2	0.4
Specialty	Medical field	268	56.7
	Non-medical specialty	205	43.3

Participants' knowledge scores on organ donation, categorized as good or poor, were analyzed across demographic and professional categories. No significant differences in knowledge were observed based on gender, nationality, region of residence, age, marital status, or educational level, as indicated by their respective p-values. In contrast, a statistically significant disparity was found in relation to working status: employees demonstrated higher knowledge scores compared to students (χ² = 6.30, p = 0.012). Opinions on organ transplantation showed no significant association with knowledge scores (Table 2).

**Table 2:** Factors affecting the knowledge score of the participants about organ donation

Variable	Category	Good knowledge	Poor knowledge	Total	χ² value	p-value
Gender	Female	129	152	281	1.86	0.173
	Male	76	116	192		
Nationality	Non-Saudi	53	72	125	0.0612	0.805
	Saudi	152	196	348		
Region of residence	Central region	58	56	114	3.76	0.44
	Eastern region	42	57	99		
	Northern region	15	23	38		
	Southern region	44	68	112		
	Western region	46	64	110	1.71	0.634
Age category	≤25	180	245	425		
	26-35	16	14	30		
	36-45	7	7	14		
	>45	2	2	4		
Marital status	Single	184	246	430	0.898	0.638
	Married	20	20	40		
	Divorced	1	2	3		
Educational level	Higher education	123	145	268	1.73	0.422
	Secondary education	81	122	203		
	Intermediate education	1	1	2		
Specialty	Medical field	126	142	268	3.4	0.065
	Non-medical specialty	79	126	205		
Working status	Student	174	247	421	6.3	0.012
	Employee	31	21	52		
Opinion on organ transplantation	I would not mind	49	72	121	1.91	0.591
	I would stand against it categorically	46	67	113		
	I would support it	51	54	105		
	I would support it under certain terms	59	75	134		

Distribution of opinions on organ transplantation among participants

**Figure 1:**
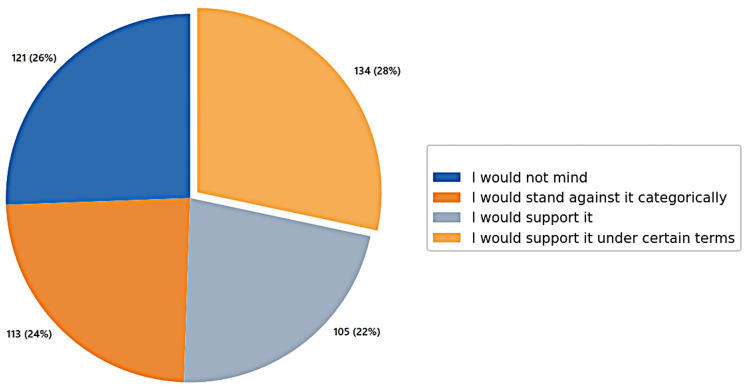
Participants' willingness for organ donation

The largest group, representing 28% (n = 134), supported organ transplantation under specific terms. This was followed by 26% (n = 121) who indicated they would not mind, 24% (n = 113) who stood against it categorically, and 22% (n = 105) who expressed full support (Figure 1).

Perceived main goals of organ transplantation among participants

**Figure 2:**
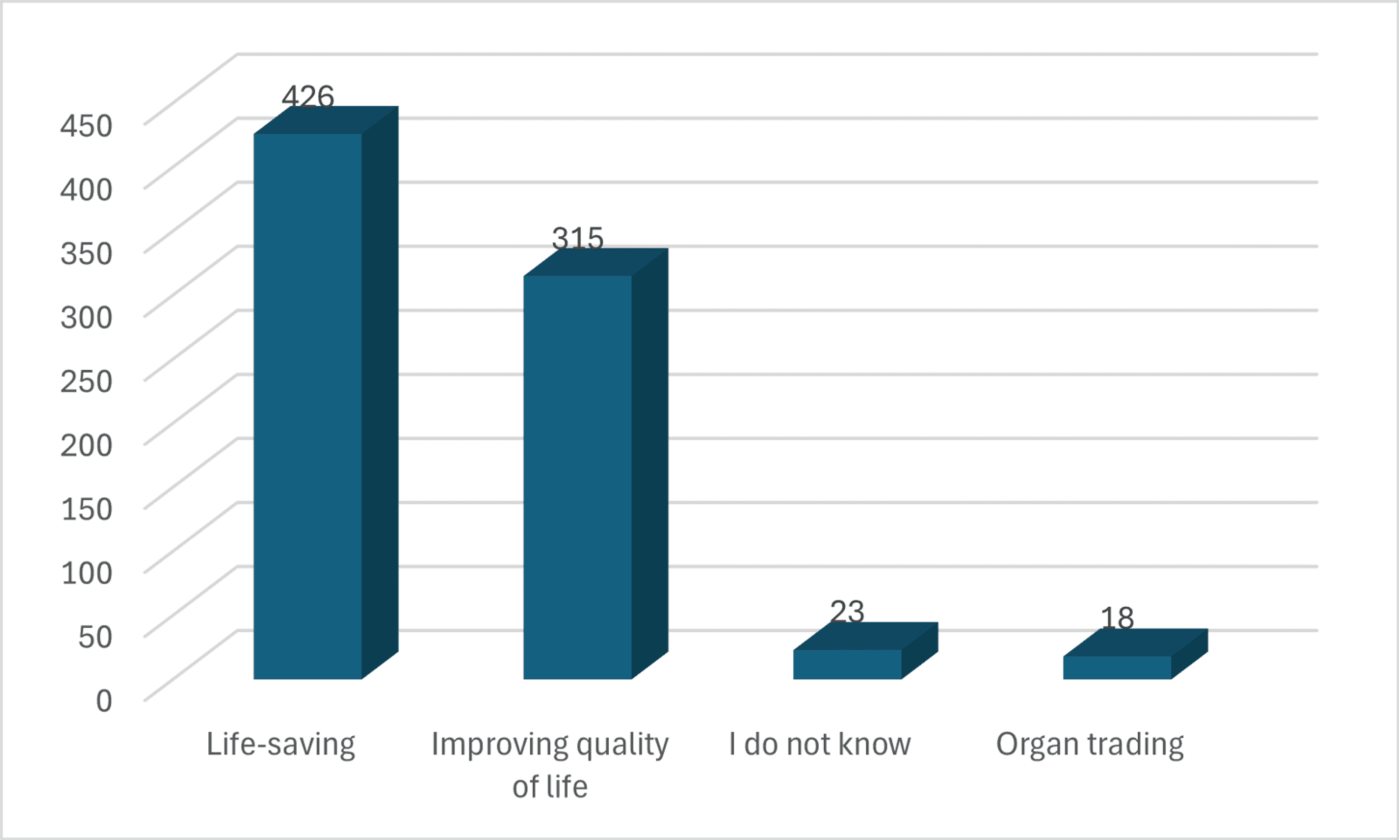
Goal of organ donation

The majority (426 (90%)) identified lifesaving as the primary purpose. A substantial proportion (314 (66%)) selected improving quality of life, while smaller groups reported "I do not know" (23 (5%)) and organ trading (10 (2%)) (Figure 2).

Participants' knowledge and perceptions of organ transplantation

Most participants recognized brain death as the total and irreversible cessation of brain functions (270 (57.1%)). The preferred recipients for living donors were anyone in need without compensation (307 (64.9%)). Organ rejection was identified as the most serious post-transplant complication (96 (20.3%)), while malignant tumors were considered the leading donor illness that makes transplantation impossible (202 (42.7%)). Additionally, a significant majority believed kidney transplant recipients can lead full lives, including sports activities (375 (79.3%)), and many expected the transplanted kidney to function for more than 10 years (321 (67.9%)) (Table 3).

**Table 3:** Participants' knowledge and perceptions on organ transplantation

Category	Subcategory	Count	% of total
Brain death definition	All of the above	84	17.80%
Living donor recipients	Cessation of breathing and circulation	17	3.60%
	Coma	61	12.90%
	I do not know	33	7.00%
	None of the above	8	1.70%
	Total and irreversible cessation	270	57.10%
	I do not know	60	12.70%
	Only for relatives	21	4.40%
	Only to close friends or loved ones	13	2.70%
	To relatives, friends, and loved ones	72	15.20%
	Anyone in need, no compensation	307	64.90%
Serious complications post-transplant	Bleeding	7	1.50%
	Infection	18	3.80%
	Organ rejection	96	20.30%
	None of the above	4	0.80%
Donor illnesses making transplantation impossible	Active infection	151	31.90%
	Benign tumors	10	2.10%
	Malignant tumors	202	42.70%
	None of the above	7	1.50%
Patient life quality post-kidney transplantation	Bedridden	17	3.60%
	Non-physical work only	64	13.50%
	Full life, may do sport	375	79.30%
	Severe impairment, unable to work	17	3.60%
Functional duration of transplanted kidney	1 year	45	9.50%
	2-3 years	107	22.60%
	More than 10 years	321	67.90%

Distribution of knowledge levels among participants concerning organ donation

**Figure 3:**
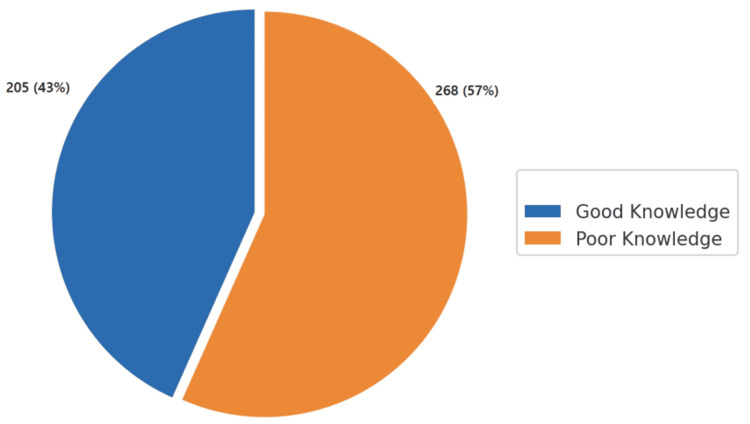
Knowledge level of participants

Good knowledge was observed in 205 (43%) participants, while poor knowledge was identified in 268 (57%) participants. Proficiency was defined by a cutoff score of 60%, with each question contributing 1 point to the total score calculation (Figure 3).

Regression analysis of the factors influencing opinions about organ transplantation after death

The baseline opinion, "I would not mind," shows minimal support with a negative coefficient (-6.2405) and an odds ratio close to zero (0.00195). Gender significantly affects attitudes; male participants are more than twice as likely to support organ transplantation as female participants, indicated by a positive coefficient (0.8155) and an odds ratio of 2.26026 (p = 0.005). Age also affects views, with a pronounced decrease in support among those aged 36-45, shown by a coefficient of -18.2032 (p < 0.001). Marital status also plays a role; divorced individuals are much less likely to support transplantation, reflected by a negative coefficient (-5.8065) and a low odds ratio (0.00301, p < 0.001). The analysis of knowledge level indicates that the baseline likelihood of poor knowledge is significant (p = 0.001) with an odds ratio of 1.595. Medical professionals are less likely to have poor knowledge compared to non-medical professionals, with a marginally significant odds ratio of 0.707 (p = 0.066) (Table 4).

**Table 4:** Model coefficients for opinions on organ transplantation after death

Predictor	Estimate	p-value	Odds ratio
Intercept (baseline: I would not mind)	-6.2405	0.719	0.00195
Gender (male versus female)	0.8155	0.005	2.26026
Age category (versus 26-35)			
36-45	-18.2032	<0.001	1.24E-08
≤25	0.1196	0.875	1.12705
Marital status (versus single)			
Divorced	-5.8065	<0.001	0.00301
Married	0.5333	0.467	1.70453
Education level (versus higher education)			
Intermediate	-2.3691	<0.001	0.09357
Secondary	-0.0481	0.868	0.95303
Knowledge of transplant cases (versus heard from media)			
No	-0.2753	0.377	0.75931
Family member	-0.6531	0.222	0.52042
In surroundings	-0.3349	0.463	0.71542
Knowledge level (predicting poor knowledge)			
Intercept	0.467	0.001	1.595
Medical field (non-medical specialty)	-0.347	0.066	0.707

## Discussion

Organ donation is still a vital but underutilized medical intervention, especially in places like Saudi Arabia, where barriers based on education, culture, and religion still exist. While the majority of participants (57%) showed adequate awareness of organ donation, our study shows that a considerable percentage (43%) showed severe gaps, especially with regard to legal and procedural factors. These results are consistent with other studies showing that, even among medical students who are supposed to promote organ donation, misunderstandings and a lack of knowledge reduce rates [11].

The fact that there are no appreciable differences in knowledge by age, gender, or level of education suggests that misleading information is widespread and not exclusive to any one group. The need for systematic educational interventions in medical courses is highlighted by the higher knowledge ratings among hired professionals as opposed to students, which may reflect experiential learning in clinical settings.

Although the difference was not statistically significant, our findings show that medical professionals had somewhat more knowledge than non-medical individuals (p = 0.066). Given that doctors are crucial in advising prospective donors and receivers, this emphasizes the need for improved organ donation education in medical school programs. Prior research has demonstrated that focused educational initiatives may greatly enhance awareness and sentiments around organ donation [12]. The fact that 64.9% of participants agreed that organs should be donated to "anyone in need" suggests a progressive mindset, but the 22% categorical rejection shows enduring opposition that may have its roots in cultural or religious reluctance. Resolving these issues via religious support and community involvement may allay fears [13].

Male participants were more than twice as likely as female participants to support organ donation, according to the regression analysis (OR = 2.26, p = 0.005). This pattern is seen in other conservative countries where gender norms play a role in medical decision-making [14]. Furthermore, older participants (36-45 years) and divorced people demonstrated significantly less support, indicating that opinions of organ donation are influenced by sociocultural variables and life experiences. These results emphasize the necessity of customized awareness programs that target issues unique to particular demographics. Furthermore, there is a persisting misunderstanding that may influence therapeutic conversations regarding deceased donation, as seen by the high recognition of the brain death criterion (57.1%) compared to the 12.9% who confused it with coma [15].

Limitation

There are many restrictions on this study. First, because the convenience sample approach was used to recruit participants mostly through social media, it may have limited the findings' generalizability by omitting people without internet access. Second, it is unable to demonstrate causal links between demographics, attitudes, and knowledge because of the cross-sectional design. Third, answer bias may affect self-reported statistics, especially when it comes to delicate subjects such as organ donation. Last, but not least, the study concentrated on medical students and a control group but did not thoroughly examine more profound cultural or religious effects, which would have shed more light on opposition to organ donation. To fill these deficiencies, future research should use qualitative techniques and stratified random sampling.

## Conclusions

This study found that Saudi Arabian medical students and professionals demonstrated moderate knowledge and generally positive attitudes toward organ donation; however, significant misconceptions persist, particularly concerning brain death, legal frameworks, and religious permissibility. Support for posthumous donation was influenced by gender and other sociodemographic factors, highlighting the role of cultural and demographic variables. To address these gaps, structured modules on organ donation should be incorporated into medical curricula, while broader public campaigns involving religious and community leaders are needed to strengthen acceptance. By tackling these deficiencies through education and awareness, future physicians will be better positioned to advocate for organ donation, improve public trust, and contribute to bridging the persistent gap between the demand and supply of transplantable organs in Saudi Arabia.
